# ADAM Metallopeptidase Domain 33 (ADAM33): A Promising Target for Asthma

**DOI:** 10.1155/2014/572025

**Published:** 2014-04-10

**Authors:** Priya Tripathi, Shally Awasthi, Peisong Gao

**Affiliations:** ^1^Johns Hopkins Asthma and Allergy Center, Johns Hopkins University School of Medicine, 5501 Hopkins Bayview Circle, Room 3B.71, Baltimore, MD 21224, USA; ^2^Department of Pediatrics, King George's Medical University, Lucknow, India

## Abstract

Over the last few years, a significant progress has been made in understanding the role of a disintegrin and metalloproteinase 33 (ADAM33) in asthma. The previous observations for the association with asthma have been replicated in over 33 different population samples worldwide. We and others have performed association analysis and meta-analysis and provided further evidence that several polymorphisms in the ADAM33 are risk factors for asthma, especially in the Asian population. Further, several studies have suggested that alterations in epigenetic marks alter the patterns of DNA methylation of ADAM33 and result in potentially adverse biological effects. Finally, while the biological activities of ADAM33 are as yet unknown, ADAM33 may play a possible role in airway remodeling because of its high expression in epithelium, myo/fibroblasts, and airway smooth muscle cells (ASMCs) and its role in promoting angiogenesis and stimulating cell proliferation and differentiation. Thus, ADAM33 represents a promising target for asthma. However, further investigations are clearly needed to discover functional ADAM33 gene polymorphisms and the role of genetic/epigenetic factors in conferring genetic susceptibility to environmental exposure induced asthma as well as biological function in asthma. This, in turn, will unlock the possibility of ADAM33 as a target for asthma therapy.

## 1. Introduction

Asthma is a complex inflammatory disorder of airways of lungs resulting in airflow obstruction and bronchial hyperresponsiveness (BHR) to a variety of stimuli and symptoms of wheeze, cough, and breathlessness. It continues to have a severe impact on global public health problem, affecting an estimated 300 million people worldwide [1]. The major obstacle in preventing and treating asthma has been our incomplete understanding of its etiology and biological mechanisms. Recent studies have changed our understanding of asthma from a purely inflammatory disease to a disease in which both inflammatory and structural components are equally involved [2]. Asthma is often associated with structural remodeling of the airways characterized by airway epithelial damage, wall thickening, and subepithelial fibrosis [2, 3]. Although environmental factors are important in the origins and progression of asthma, it is widely recognized that asthma has a strong genetic component and is the result of complex interactions between genes and environment [3–5]. In the last decade, tremendous progress has been made in the genetic study of asthma with many genes identified as asthma-susceptible genes. Of these, a disintegrin and metalloproteinase 33 (ADAM33) gene is the first novel susceptibility gene for asthma and airway hyperresponsiveness (AHR) identified by positional cloning [6] and has been replicated in over 33 different population samples worldwide [7]. We and others have recently performed meta-analysis and provided further evidence that several polymorphisms in the ADAM33 are risk factors for asthma, especially in the Asian population. Although the biological activities of ADAM33 remain unknown, we speculate that ADAM33 might be associated with airway remodeling because of its high expression in airway fibroblasts, myofibroblasts, and smooth muscle cells and its function in protecting the airway from increased repair processes [8]. In this paper, we reviewed the studies on ADAM33, including replication of associations and meta-analysis between ADAM33 polymorphisms from the original studies and asthma and related phenotypes in different populations, particularly in the Asian populations, epigenetic mechanisms for ADAM33 in asthma, and possible biologic link to the pathogenesis of asthma.

## 2. Association of ADAM33 Gene Polymorphisms with Asthma and Related Phenotypes

**Figure 1:**
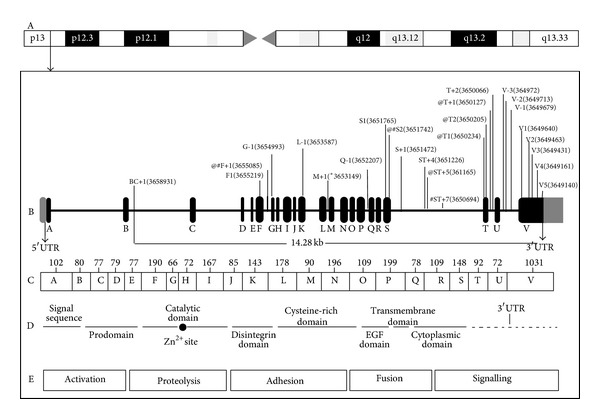
Schematic representation of the ADAM33 gene on chromosome 20. (a) Chromosome 20 showing ADAM33 gene position on 20p13. (b) Region covered by all most common studied polymorphisms by study groups in worldwide population and covered size in Kb. (c) Exons and size in base pairs. (d) Domain structure. (e) Functions of ADAM33 domain. @ represents positive association among Asians; # represents positive association among Caucasians.

The first asthma-susceptibility locus to be identified by positional cloning was reported by Van Eerdewegh et al. A genomewide scan in 480 asthma sibling-pair families from the UK and US revealed an evidence for linkage between asthma and BHR on chromosome 20p13 (Figure 1), where ADAM33 is located and associated with asthma [6]. ADAM33 belongs to members of disintegrin and metalloprotease family that code for zinc-dependent metalloproteases. It is a type I transmembrane zymogen glycoprotein. The ADAM33 protein harbors several domains that include pro-metalloprotease-like, disintegrin-like, cysteine-rich, epidermal growth factor-like, transmembrane, and cytoplasmic domains facilitating its participation in many cellular processes [9–12]. Its adhesion domain as well as protease domain makes it exclusive among cell surface proteins. The autocatalytic removal of the prodomain is activation signal for ADAM proteins [12]. ADAM33 is a complex molecule whose expression is restricted largely to mesenchymal cells including airway fibroblasts, myofibroblasts, and smooth muscle cells [6, 13]. Figure 1 is the schematic representation of the ADAM33 gene on chromosome 20, including the ADAM33 gene location (A), all most common studied polymorphisms by study groups in worldwide populations (B). ADAM33 includes 22 exons size in base pairs (C) with different genomic domains (D), which contribute to several important biological functions of the ADAM33 gene, including cell activation, proteolysis, adhesion, fusion, and signaling (E).

After Van Eerdewegh et al.'s study, a large number of studies have been carried out. For instance, Howard and colleagues assessed 8 selected ADAM33 SNPs from the original study and provided further evidence of association with asthma in four case-control asthma populations—African American (AA), US Caucasian, US Hispanic, and Dutch Caucasian [14]. While association was identified for at least one SNP in each population, none of SNPs was seen to be associated with asthma phenotype across all those studied populations. Association with asthma was also found in family-based studies. Werner et al. analyzed 15 highly selected ADAM33 SNPs in 171 German families and found that SNPs F+1, ST+4, and ST+5 were associated with asthma [15]. Several other reports demonstrated that ADAM33 polymorphisms have shown association with decline in FEV1 in asthmatic adults [16] and impaired lung function at ages 3 and 5 years [17], respectively. In contrast, few studies failed to replicate the association including a study in 583 Hispanic (Puerto Rican and Mexican) trios [18] and a study in a large population of North American asthma trios [19]. In Asian populations, the associated ADAM33 SNPs were different. Chi and his colleagues reported that SNP S2 was associated with risk of asthma in the Chinese Han population [20]. However, in the same population, Jie et al. failed to find association for S2 but found significant association for SNPs F+1, T1, and T2 [21]. In a Thai population Thongngarm et al. found that SNP S2 and ST+4 were associated with asthma susceptibility [22]. We conducted a study in an Indian population and assessed association for 14 ADAM33 SNPs (F+1, V4, ST+4, S2, ST+5, V2, T2, T1, BC+1, Q-1, S1, S+1, V-3, and T+1) and found 8 SNPs (F+1, V4, ST+4, S2, ST+5, T2, T1, and S1) in association with asthma [23–26]. In Supplementary Table 1 (see Supplementary Material available online at http://dx.doi.org/10.1155/2014/572025), we listed a total of 26 studies extracted from PubMed of different groups of populations. While studies have suggested that ADAM33 gene polymorphisms are important in conferring susceptibility to asthma, the data are controversial. Moreover, the true causative variant that determines asthma predisposition is still unclear. Furthermore, many of these individual studies had a relatively smaller sample size, which are statistically underpowered. Finally, it is worth noting that environmental exposure may modulate the genetic associations observed in different populations (gene-environment interaction). Indeed, Reijmerink et al. confirmed that interaction of in utero cigarette smoke exposure with ADAM33 results in reduced lung function and the development of BHR [27]. Similarly, we also observed enhanced association of ADAM33 SNP V4 heterozygous with reduced FEV1 among smokers [28].

**Figure 2:**
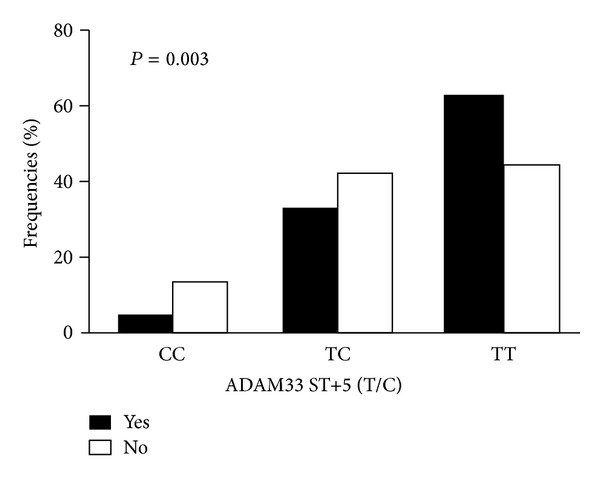
Environmental exposure modulates the association for ADAM33 ST+5 and asthma.* x-*axis represents ST+5 genotypes; *y*-axis represents frequencies of genotype in individuals with (yes) or without (no) exposure to heavy traffic.

Very recently, we observed that one of asthma associated ADAM33 ST+5 was significantly associated with heavy traffic, an indication of air pollution, in a total of 386 Indian individuals. The heavy traffic was defined based on the distance from heavy traffic [29]. We have found that the heavy traffic-predicted percentages for the homozygous normal genotype (CC), the heterozygous genotype (TC), and the homozygous mutant genotype (TT) of ST+5 SNP were 4.4, 33.3, and 62.2%, respectively. In contrast, nontraffic area predicted percentages for CC, TC, and TT of ST+5 SNP were 13.5, 42.6, and 43.9%, respectively (OR = 1.934, 95%  CI = 1.306–2.863, and *P* = 0.001; Figure 2), indicating that TT genotype may protect against heavy traffic. These findings suggest that exposure to environmental chemicals, together with genetic variations, may contribute to the increased risk of asthma.

## 3. ADAM33 Gene Polymorphisms and Risk of Asthma: A Meta-Analysis

In the association studies, there are possibilities that some positive results might be specious and some negative findings might be a consequence of low statistical power. It could be due to their smaller sample size or methodological liabilities, such as the selection of an appropriate control group. Meta-analysis might be a means of determining reflective results. Conceptually, a meta-analysis uses a statistical approach to combine the results from multiple studies [30]. It has been widely used for genetic association analysis by combining the results from different studies with a relatively larger sample size. Specifically, combining samples from several studies could make greater power than from individual studies or might increase trends for association in small individual studies. Further, meta-analysis might be useful to identify the causative gene polymorphisms with consistency and to quantify with accuracy the genetic risks. Thus, in this review, we summarized the major meta-analyses to see whether these ADAM33 SNPs were associated with asthma in an increased sample size, including our meta-analysis in the Asian population [31].

**Figure 3:**
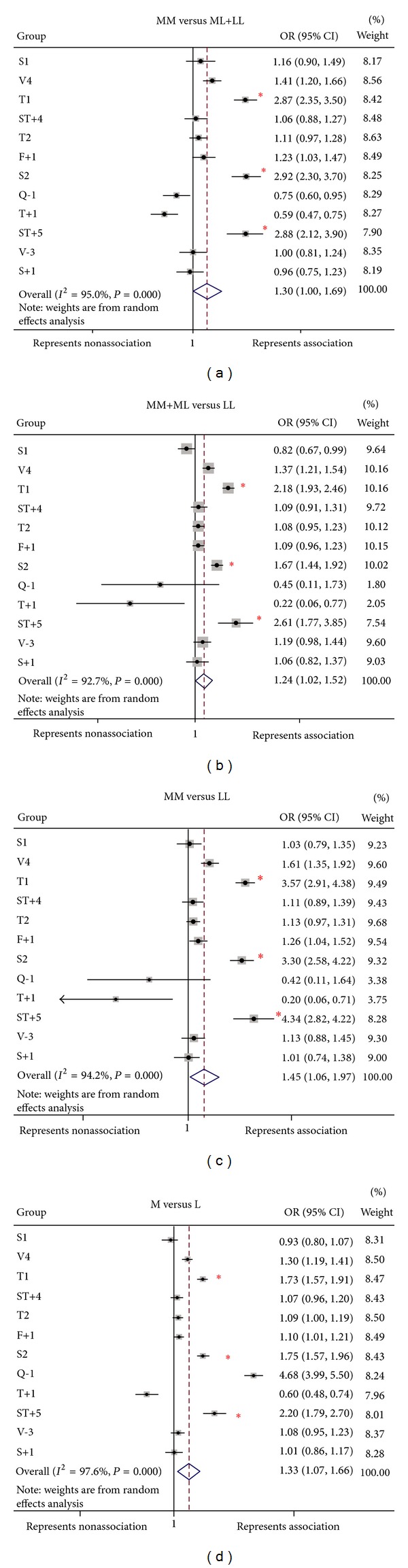
Forest plots from my meta-analysis study on Asian population. (a) MM versus LL+LM, (b) MM+ML versus LL, (c) MM versus LL, and (d) M versus L, where M is mutant allele, L is wild-type allele, LL is homozygous normal genotype, LM is heterozygous genotype, and MM is homozygous mutant genotype. *Significant associations with odds ratios from 1.67 to 4.34 in the overall population under different models.

In our study, we reviewed 13 studies on 12 ADAM33 polymorphisms in Asian populations and quantitatively summarized the association between ADAM33 SNPs (S1, V4, T1, ST+4, T2, F+1, S2, Q-1, T+1, ST+5, V-3, and S+1) and asthma using meta-analysis. A dominant (MM+ML versus LL), recessive (MM versus ML+LL), additive (MM versus LL), and allelic models (M versus L) were used to estimate the association between ADAM33 gene polymorphisms and asthma risk in a total of 6212 individuals consisting of 3,270 patients and 2,922 controls. Significant associations were found for ST+5, S2, and T1 with odds ratios from 1.67 to 4.34 in the overall population [31] (Figure 3). The evidence from the meta-analyses supports the notion of a role for the SNPs ST+5, S2, and T1 in the ADAM33 gene in conferring susceptibility to asthma in the Asian population. Very recently, Liang et al. performed similar meta-analysis to summarize the associations between ADAM33 polymorphisms and asthma risk for a total of 29 case-control studies and 14 SNPs. Although they failed to find association for ADAM33 SNPs S1, V-1, V5, S+1, S2, ST+4, ST+7, ST+5, and Q-1, significant associations were observed for T1, V4, F+1, and T+1 in the overall population. Interestingly, a positive result was found for the T1, V4, F+1, and T2 polymorphisms only in Asia but not in Europe or Latin America. This meta-analysis provides further evidence that the T1, V4, F+1, and T2 in the ADAM33 gene may be the major risk factors for asthma, especially in the Asian population [32]. Another meta-analysis performed by Song et al. included thirteen studies in ten reports for the association between the ADAM33 SNPs and asthma in a total of 12,875 subjects consisting of 4942 patients and 7933 controls. Song et al.'s group was limited to selection of SNPs. This group selected only five ADAM33 SNPs, named as S2, ST+4, F+1, S1, and V4. They found that the ADAM33 SNP S2 confers susceptibility to asthma in Europeans and ST+4 in Asians and adults [33]. Taken together, these meta-analyses suggest that the ADAM33 SNPs confers susceptibility to asthma. Different ethnic populations showed the associations with different SNPs, which may be in linkage disequilibrium with the same causal variants in ADAM33. Further studies are required to identify functional mutations, which may modulate the ADAM33 function and subsequently development of asthma. Meta-analysis results also highlight the fact that no single SNP is associated with asthma across all the populations studied. This may be due to the compound effects of multiple alleles, multiple genes, and environmental factors. For example, the effect of a given SNP could be restricted to an ethnic group or a population; thus, there may be genetic differences among subtypes of asthma, such as pediatric, adult, and allergic/nonallergic. Secondly, this could be due to the difference in the exposure to allergens, which could vary from one geographical region to another and their interactions with the genetic factor(s) could vary. Thirdly, the linkage disequilibrium patterns that exist between the identified SNP and the undetected causative defect in the gene could differ from one population to another [34]. Thus, through understanding the genetic factors and their interactions with the environmental factors for each population, this would aid in developing effective predictive markers for the multifactorial diseases like asthma.

## 4. Epigenetic Mechanisms for ADAM33 in Asthma

Several studies have suggested that the pathogenesis of asthma may be affected by epigenetic regulation. So far, there have been 4 studies on the ADAM33 DNA methylation and asthma [35–38]. Yang et al. investigated the methylation status of CpG island within its promoter of ADAM33, and its association with ADAM33 expression. Interestingly, they found that the CpG site in the promoter (−362 to +80) of ADAM33 was hypermethylated in epithelial cells but hypomethylated in ADAM33-expressing fibroblasts [35] and suggested that the methylation status controls ADAM33 expression in a cell-type-specific manner. Studies from the same group reported that no changes were observed in methylation status of the ADAM33 promoter in normal or asthmatic fibroblasts. However, they found that transforming growth factor-beta 2 (TGF-*β*2) downregulates ADAM33 mRNA expression in normal and asthmatic fibroblasts by causing chromatin condensation around the ADAM33 promoter with deacetylation of histone H3, demethylation of H3 on lysine-4, and hypermethylation of H3 on lysine-9. Findings from this study suggested that TGF-*β*2 suppresses expression of ADAM33 mRNA expression by chromatin modification, rather than by gene silencing through DNA methylation [36]. This was further supported by a very recent study by investigating the methylation patterns of ADAM33 in adult asthma [37]. They designed a case-control study with 50 asthmatic patients and 50 age- and sex-matched healthy controls to examine the relationship between the CpG methylation of the ADAM33 gene and asthma using bisulfite deoxyribonucleic acid modification and sequencing. A total of 14 CpG sites in exon 9 of the ADAM33 gene were found to be highly methylated (100%) in all individuals, but no clear difference in DNA methylation patterns between asthmatic and controls groups was shown. The negative observation may be due to the limited sample size and confounding factors like environmental exposures. Alterations in epigenetic marks have been associated with exposures relevant to asthma, particularly air pollution and tobacco smoke, as well as asthma phenotypes [38]. We postulate that exposure to environmental chemical or allergens can alter the patterns of DNA methylation of ADAM33, resulting in potentially adverse biological effects such as aberrant gene expression and ADAM33-involved airway remodeling. Further investigations are needed to examine the epigenetic changes of ADAM33 in a larger population with or without the considerations of environmental factors. Thus, epigenetic mechanisms represent a promising direction that might, in part, explain the inheritance and immunobiology of asthma.

## 5. Biological Link of ADAM33 to Asthma

**Figure 4:**
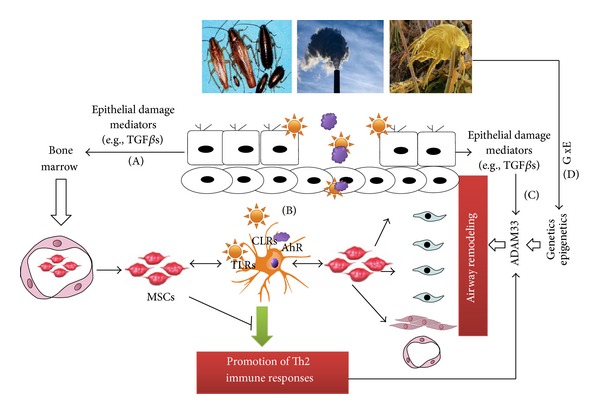
Schematic representation of possible mechanisms for ADAM33 in airway remodeling.

ADAM33 has been identified as an asthma susceptibility gene; however, the role of ADAM33 in the pathogenesis and progression of asthma remains to be elucidated. ADAM33 is predominantly expressed in cells of mesenchymal origin, mainly as fibroblasts, myofibroblasts, and smooth muscle cells, indicating a possible role in airway remodeling (see Figure 4) [6, 13, 39]. Indeed, increased expression of ADAM33 was detected in human airway from subjects with asthma as compared to that in controls. Further, the increased expression was correlated with asthma severity progressed from mild to severe and with lung function as defined by FEV1% [40, 41]. We investigated the expression of ADAM33 protein in bronchial biopsy tissues from 27 patients with asthma and 7 nonasthmatic controls [42]. We, for the first time, identified increased expression of ADAM33 protein in epithelium, smooth muscle and mesenchymal cells of asthmatic patients when compared to those nonasthmatic controls. Given the small sample size, we failed to find a correlation between ADAM33 expression and severity of asthma. As ADAM33 is predominantly expressed in airway smooth muscle cells (ASMCs), Lin et al. investigated whether ADAM33 protein expression is correlated with ASMC mechanics in an ovalbumin- (OVA-) sensitized rat model. Increased ADAM33 expression was observed in ASMCs from the OVA-sensitized rats when compared with the nonsensitized rats. Importantly, ADAM33 expression was positively correlated with cell stiffness, traction force, and expression of vinculin and Factin, suggesting that ADAM33 is a mediator of ASMC dysfunction in asthma [43]. These increased expression of ADAM33 in epithelium in asthmatic patients and ASMCs in allergen-induced animal models further support the possibility that ADAM33 may play a role in airway remodeling.

Although the mechanisms for the ADAM33 involved remodeling are not clear, it has been suggested that ADAM33 may affect the epithelial-mesenchymal trophic unit (EMTU) [35, 44]. Moreover, the soluble form of ADAM33 causes rapid induction of endothelial cell differentiation in vitro and neovascularization ex vivo and in vivo, suggesting that ADAM33 can promote angiogenesis and lead to airway remodeling. TGF*β* is a multifunctional cytokine that plays a critical role in cell growth, differentiation, and immune regulation and has been considered a principal mediator of airway remodeling [45–48]. Recent studies have demonstrated that disruption in TGF*β*1 signaling imposes a strong predisposition for human allergic diseases [49]. Specifically, increased active TGF*β*1 has been observed in airways from asthmatic patients [50] and from experimental mice during allergic airway inflammation [51]. Because TGF*β*1 can promote ADAM33 ectodomain shedding and suppress ADAM33 expression, it is possible that active TGF*β*1 may cause fibroblast differentiation and proliferation through regulating ADAM33 expression and subsequently lead to airway remodeling. As summarized in Figure 3, we postulate that (1) active TGF*β*1 released from damaged/repairing epithelium in response to repetitive allergen and environmental chemical challenges may cause aberrant excessive recruitment of mesenchymal stem cells (MSCs), leading to accumulation of fibroblasts/myofibroblasts and progressive fibrosis and pathological remodeling due to their capability of differentiation into myofibroblasts [52]; ADAM33 may be required for this process. (2) these released active TGF*β*1 can directly modulate ADAM33 expression and subsequently functions; (3) allergens and environmental chemicals can disturb airway epithelial integrity and lead to an increased penetration of allergens and chemicals, resulting in activation of innate immune cells (e.g., dendritic cells (DCs)) through C-type lectin receptors (CLRs), Toll-like receptors (TLRs), and aryl hydrocarbon receptor (AhR), which will direct cells of the adaptive immune system to Th2 cell development with the upregulation of ADAM33 mRNA expression [53]; and (4) environmental factors, together with genetic or epigenetic changes of ADAM33 (interaction between environmental and genetic factors), may cause aberrant expression of ADAM33 in epithelial, fibroblasts, and airway smooth muscle cells. It is possible that all of these may synergize, leading to the development of airway remodeling.

## 6. Conclusions

In this paper, we have reviewed the studies on the replications and meta-analysis of genetic associations for ADAM33 and asthma and its related phenotypes in different populations. While studies, including association studies and meta-analyses, have suggested that several ADAM33 gene polymorphisms (e.g., ST+5, S2, and T1) are important in conferring susceptibility to asthma, the data are controversial, and the true causative variants have not been identified yet. Many factors may be related to the failure to identify the true associations and causal variants, including sample size, environmental modification (interplay between genetic and environmental factors), population mixture, type of clinical asthma, age of onset, and treatment history. Thus, larger scale studies in different ethnic, but age-, sex-, and environmental exposure-matched populations are required to continue these studies. Further, there are few, but important, studies on investigating the effects of on the epigenetic modulation on ADAM33 function. Alterations in epigenetic marks have been shown to alter the patterns of DNA methylation of ADAM33, resulting in potentially adverse biological effects. Thus, epigenetic mechanisms represent a promising direction for the studies on the development of asthma. Finally, while the biological activities of ADAM33 are as yet unknown, it has been suggested that ADAM33 may play a possible role in airway remodeling because of its expression in epithelium, myo/fibroblasts, and ASMCs and its role in promoting angiogenesis and stimulating cell proliferation and differentiation. It is likely that ADAM33 mediates environmental exposure induced airway inflammation and remodeling that may occur through the activation of TGF*β* signaling and several major receptors (CLRs, AhR, and TLRs). Thus, ADAM33 represents a promising target for asthma. However, further studies are clearly needed to discover functional SNPs, the role of genetic/epigenetic factors in conferring genetic susceptibility environmental exposure induced asthma, and biological function in asthma.

## Supplementary Material

We listed the ADAM33 gene polymorphisms in a total of 26 different studies extracted from PubMed.
