# Supplementary material for: ADAM Metallopeptidase Domain 33 (ADAM33): A Promising Target for Asthma
Source: Mediators Inflamm. 2014 Apr 10;2014:572025. doi: 10.1155/2014/572025 (PMC4003756; doi:10.1155/2014/572025)
Supplement: Supplementary file 1 — We listed the ADAM33 gene polymorphisms in a total of 26 different studies extracted from PubMed. [file 572025.f1.pdf]

**Supplementary Table: 1 Summary of studied ADAM33 polymorphisms in various studied populations**

| Ref. | Study Population | N | ADAM33 SNPs and reported association | | | | | | | | | | | | | | | | | | | |
|---|---|---|---|---|---|---|---|---|---|---|---|---|---|---|---|---|---|---|---|---|---|---|
| | | | T1 | T2 | S1 | Q-1 | BC+1 | V2 | F+1 | S2 | ST+4 | ST+5 | V4 | S+1 | V-3 | T+1 | ST+7 | V-1 | F1 | G-1 | L-1 | M+1 |
| 1-4 | India(cc) | 386/390 | + | + | + | — | — | — | + | + | + | + | + | — | — | — | * | * | * | * | * | * |
| 5 | Chez Republic(cc) | 109/46 | — | * | — | * | * | * | — | — | — | * | — | — | — | — | * | * | * | * | — | * |
| 6 | Saudi Arabia (cc) | 107/87 | + | + | — | * | * | * | * | * | — | * | * | * | * | * | * | * | * | * | * | * |
| 7 | Japanese women(cc) | 89/1281 | — | — | * | — | * | * | * | — | * | * | — | — | — | — | * | * | * | * | * | * |
| 8 | Czech Republic(cc) | 109/45 | — | * | — | * | * | * | — | — | — | * | — | — | — | — | * | * | * | * | — | * |
| 9 | Chinese (cc) | 329/316 | * | — | * | * | * | * | — | + | * | * | — | * | * | * | * | * | * | * | * | * |
| 10 | Northern Chinese(cc) | 412/397 | + | — | * | — | * | * | — | * | * | * | — | * | * | + | * | * | * | * | * | * |
| 11 | East China Han Population(cc) | 150/74 | + | + | — | * | * | * | + | — | — | * | * | * | * | * | * | * | * | * | * | * |
| 12 | Cartagena, Colombia(cc) | 429/401 | — | — | * | * | * | * | * | — | * | * | — | * | * | * | * | — | * | * | * | * |
| | Cartagena, Colombia(fa) | 116(348) | — | — | * | * | * | * | * | — | * | * | — | * | * | * | * | — | * | * | * | * |
| 13 | Chinese Han (cc) | 181/151 | + | + | — | + | * | * | * | * | * | * | + | * | * | — | * | * | * | * | * | * |
| 14 | Japanese (cc) | 101/120 | — | — | * | * | * | * | * | * | — | * | — | * | — | — | + | + | * | * | * | * |
| | | 282/120 | — | — | * | * | * | * | * | * | — | * | — | * | — | — | — | — | * | * | * | * |
| 15 | Japanese (fa) | 155(538) | — | + | — | — | * | — | — | — | + | * | — | + | — | — | — | — | — | — | — | — |
| 16 | Australian (cc) | 612/473 | * | * | — | — | * | — | — | * | — | * | — | * | * | * | — | — | * | * | * | * |
| 17 | Chinese(cc) | 296/270 | — | * | * | * | * | * | — | * | * | * | * | — | * | * | * | * | * | * | * | * |
| 18 | German(cc) | 624/1248 | — | — | — | * | * | * | — | — | — | — | — | * | * | * | — | * | * | * | * | — |
| | Cohort (coh) | 86/464 | — | — | — | * | * | * | — | — | — | — | — | * | * | * | — | * | * | * | * | — |
| 19 | Japanese (cc) | 504/651 | + | + | * | * | * | * | — | + | — | * | — | * | + | * | * | * | * | * | * | * |
| 20 | Icelandic (cc) | 348/262 | — | — | — | — | * | * | — | — | — | — | — | * | * | — | — | — | * | * | * | — |
| | Nottingham (fa) | 60(240) | — | — | — | — | * | * | — | — | — | — | — | * | * | — | — | — | * | * | * | — |
| 21 | Korean (cc) | 326/121 | — | * | — | * | * | * | * | * | * | * | — | * | * | * | * | — | * | * | * | * |
| 22 | Non-Hispanic white (fa) | 474(1462) | — | — | * | * | * | * | * | — | — | — | — | * | * | * | * | — | * | * | * | — |
| | Hispanic (fa) | 47(149) | + | — | * | * | * | * | * | — | — | — | — | * | * | * | * | — | * | * | * | — |
| | African American (fa) | 66(203) | — | — | * | * | * | * | * | — | — | — | — | * | * | * | * | — | * | * | * | — |
| 23 | German(cc) | 48/499 | — | — | — | — | * | * | — | — | — | — | — | — | * | — | + | — | * | * | * | — |
| | German (fa) | 171(732) | — | — | — | — | * | * | + | + | + | + | — | — | * | — | — | — | * | * | * | — |
| 24 | African American (cc) | 161/265 | — | — | — | * | * | * | * | + | — | — | — | * | * | * | — | — | * | * | * | * |
| | US White (cc) | 220/229 | + | + | — | * | * | * | * | + | — | — | — | * | * | * | + | — | * | * | * | * |
| | US Hispanic (cc) | 113/127 | + | + | — | * | * | * | * | + | — | — | — | * | * | * | — | — | * | * | * | * |
| | Dutch White (cc) | 180/133 | — | — | — | * | * | * | * | — | — | — | + | * | * | * | — | — | * | * | * | * |

| | | | | | | | | | | | | | | | | | | | | | | |
|---|---|---|---|---|---|---|---|---|---|---|---|---|---|---|---|---|---|---|---|---|---|---|
| 25 | Mexican (cc) | 190/160 | — | — | — | * | * | * | * | * | — | — | — | * | * | * | * | — | * | * | * | * |
| | Puerto Rican (cc) | 183/165 | — | — | — | * | * | * | * | * | — | — | — | * | * | * | * | — | * | * | * | * |
| | Mexican/P. Rican (fa) | 583(1749)[3] | — | — | — | * | * | * | * | * | — | — | — | * | * | * | * | — | * | * | * | * |
| 26 | US/UK combined (cc) | 130/217 | * | * | + | + | * | * | * | * | + | + | * | * | * | — | + | + | * | * | * | * |
| | UK (cc) | (not reported) | * | * | + | + | * | * | + | + | + | * | + | * | * | * | * | + | * | * | * | * |
| | US (cc) | (not reported) | + | + | * | * | * | * | * | * | * | * | * | * | * | + | * | * | * | * | + | + |
| | US/UK (fa) | 460(1840) | — | * | + | * | * | * | * | * | * | * | — | * | * | * | * | — | * | * | * | * |

**Abbreviations: cc, case- control; coh, cohort study; fa, family study; “+” association with asthma; “—”not significantly; *= not reported.**

## REFERENCES


1. Awasthi S, Tripathi P, Ganesh S, Husain N. Association of ADAM33 gene polymorphisms with asthma in Indian children. J Hum Genet, 2011; 56: 188-95.
2. Awasthi S, Tripathi P, Prasad R, Awasthi R Ganesh S. Association of ADAM33 gene polymorphisms and their haplotypes with asthma in an Indian population. Journal: Indian journal of medical sciences- Accepted
3. Tripathi P, Awasthi S, Prasad R, Husain N, Ganesh S. Association of ADAM33 gene polymorphisms with adult-onset asthma and its severity in an Indian adult population. J Genet, 2011; 90:265-73
4. Tripathi P, Awasthi S, Prasad R, Ganesh S. Haplotypic association of ADAM33 (T+1, S+1 and V - 3) gene variants in genetic susceptibility to asthma in Indian population. Ann Hum Biol. 2012 Nov-Dec;39(6):479-83.
5. Kopriva F, Godava M, Markova M, Vodicka R, Dusek L, Muzik J, Schneiderova E, Vrtel R, Mihal V. Possible control of paternal imprinting of polymorphisms of the ADAM33 gene by epigenetic mechanisms and association with level of airway hyperresponsiveness in asthmatic children. Biomed Pap Med Fac Univ Palacky Olomouc Czech Repub. 2013 Apr 23. doi: 10.5507/bp.2013.025. [Epub ahead of print]
6. Al-Khayyat AI, Al-Anazi M, Warsy A, Vazquez-Tello A, Alamri AM, Halwani R, Alangari A, Al-Frayh A, Hamid Q, Al-Muhsen S. T1 and T2 ADAM33 single nucleotide polymorphisms and the risk of childhood asthma in a Saudi Arabian population: a pilot study. Ann Saudi Med. 2012 Sep-Oct;32(5):479-86. doi: 10.5144/0256-4947.2012.479.
7. Miyake Y, Tanaka K, Arakawa M. ADAM33 polymorphisms, smoking and asthma in Japanese women: the Kyushu Okinawa Maternal and Child Health Study. Int J Tuberc Lung Dis. 2012 Jul;16(7):974-9

8. Godava M, Kopriva F, Bohmova J, Vodicka R, Dusek L, Cvanova M, Muzik J, Markova M, Schneiderova E, Vrtel R.
Association of STAT6 and ADAM33 single nucleotide polymorphisms with asthma bronchiale and IgE level and its possible epigenetic background. Biomed Pap Med Fac Univ Palacky Olomouc Czech Repub. 2012 Sep;156(3):236-47. doi: 10.5507/bp.2012.009. Epub 2012 Jan 30.
9. Chi X, Wang L, Wang J, Li Q, Wang X, Wang J, Xiao W. Association of ADAM33 gene polymorphisms with asthma in a Chinese population. Clin Respir J. 2013 Jan;7(1):16-20.
10. Qu S, Sun D, Wang Y, Zhang C, Lv Y, Yao L. Association of ADAM33 polymorphisms with childhood asthma in a northern Chinese population. *Exp Mol Pathol*, 2011; 91:775-9.
11. Jie Z, Hu Z, Bai C, Jin M. ADAM33 gene polymorphisms associate with asthma susceptibility and severity in East China Han population.J Asthma, 2011; 48:979-85.
12. Vergara CI, Acevedo N, Jiménez S, et al. A Six-SNP haplotype of ADAM33 is associated with asthma in a population of Cartagena, Colombia. *Int Arch Allergy Immunol*, 2010;152:32-40.
13. Su D, Zhang X, Sui H, Lü F, Jin L, Zhang J. Association of ADAM33 gene polymorphisms with adult allergic asthma and rhinitis in a Chinese Han population. *BMC Med Genet*, 2008; 9: 82– 87.
14. Sakagami T, Jinnai N, Nakajima T, Sekigawa T, Hasegawa T, Suzuki E, Inoue I, Gejyo F.ADAM33 polymorphisms are associated with aspirin-intolerant asthma in the Japanese population. J Hum Genet. 2007;52(1):66-72. Epub 2006 Oct 24.
15. Noguchi E, Ohtsuki Y, Tokunaga K, Yamaoka-Sageshima M, Ichikawa K, Aoki T, Shibasaki M, Arinami T. ADAM33 polymorphisms are associated with asthma susceptibility in a Japanese population. Clin Exp Allergy. 2006 May;36(5):602-8.

16. Kedda MA, Duffy DL, Bradley B, O'Hehir RE, Thompson PJ. ADAM33 haplotypes are associated with asthma in a large Australian population. Eur J Hum Genet. 2006 Sep;14(9):1027-36. Epub 2006 Jun 14.
17. Wang P, Liu QJ, Li JS, Li HC, Wei CH, Guo CH, Gong YQ. Lack of association between ADAM33 gene and asthma in a Chinese population. Int J Immunogenet. 2006 Aug;33(4):303-6.
18. Schedel M, Depner M, Schoen C, et al. The role of polymorphisms in ADAM33, a disintegrin and metalloprotease 33, in childhood asthma and lung function in two German populations. *Respir Res,* 2006; 7: 91
19. Hirota T, Hasegawa K, Obara K, Matsuda A, Akahoshi M, Nakashima K, Shirakawa T, Doi S, Fujita K, Suzuki Y, Nakamura Y, Tamari M. Association between ADAM33 polymorphisms and adult asthma in the Japanese population. Clin Exp Allergy. 2006 Jul;36(7):884-91.
20. Blakey J, Halapi E, Bjornsdottir US et al. Contribution of ADAM33 polymorphisms to the population risk of asthma. *Thorax,* 2005; 60:274-6.
21. Lee JH, Park HS, Park SW, Jang AS, Uh ST, Rhim T, Park CS, Hong SJ, Holgate ST, Holloway JW, Shin HD. ADAM33 polymorphism: association with bronchial hyper-responsiveness in Korean asthmatics. Clin Exp Allergy. 2004 Jun;34(6):860-5.
22. Raby BA, Silverman EK, Kwiatkowski DJ, Lange C, Lazarus R, Weiss ST. 2004.ADAM33 polymorphisms and phenotype associations in childhood asthma. J Allergy Clin Immunol, 2004; 113:1071-8.
23. Werner M, Herbon N, Gohlke H, Altmüller J, Knapp M, Heinrich J, Wjst M. Asthma is associated with single-nucleotide polymorphisms in ADAM33. Clin Exp Allergy. 2004 Jan;34(1):26-31.

24. Howard TD, Postma DS, Jongepier H, et al. Association of a disintegrin and metalloprotease 33 (ADAM33) gene with asthma in ethnically diverse populations. J Allergy Clin Immunol. 2003; 112:717-22.

25. Lind DL, Choudhry S, Ung N et al. ADAM33 is not associated with asthma in Puerto Rican or Mexican populations. Am J Respir Crit Care Med, 2003; 168: 1312-6.

26. Van Eerdewegh P, Little RD, Dupuis J, et al. Association of the ADAM33 gene with asthma and bronchial hyperresponsiveness. Nature, 2001; 418: 426-30.
